# Effect of continuous aerobic exercise on endothelial function: A systematic review and meta-analysis of randomized controlled trials

**DOI:** 10.3389/fphys.2023.1043108

**Published:** 2023-02-10

**Authors:** Xifeng Tao, Yiyan Chen, Kai Zhen, Shiqi Ren, Yuanyuan Lv, Laikang Yu

**Affiliations:** ^1^ Key Laboratory of Physical Fitness and Exercise, Ministry of Education, Beijing Sport University, Beijing, China; ^2^ Department of Sports Performance, Beijing Sport University, Beijing, China; ^3^ China Institute of Sport and Health Science, Beijing Sport University, Beijing, China

**Keywords:** continuous aerobic exercise, endothelial function, FMD, systematic review, meta-analysis

## Abstract

**Background:** Current research suggests that continuous aerobic exercise can be effective in improving vascular endothelial function, while the effect between different intensities and durations of exercise is unclear. The aim of this study was to explore the effect of different durations and intensities of aerobic exercise on vascular endothelial function in different populations.

**Methods:** Searches were performed in PubMed, Web of Science, and EBSCO databases. We included studies that satisfied the following criteria: 1) randomized controlled trials (RCTs); 2) including both an intervention and control group; 3) using flow-mediated dilation (FMD) as the outcome measure; and 4) testing FMD on the brachial artery.

**Results:** From 3,368 search records initially identified, 41 studies were eligible for meta-analysis. There was a significant effect of continuous aerobic exercise on improving flow-mediated dilation (FMD) [weighted mean difference (WMD), 2.55, (95% CI, 1.93–3.16), *p <* 0.001]. Specifically, moderate-intensity [2.92 (2.02–3.825), *p* < 0.001] and vigorous-intensity exercise [2.58 (1.64–3.53), *p* < 0.001] significantly increased FMD. In addition, a longer duration [<12 weeks, 2.25 (1.54–2.95), *p* < 0.001; ≥12 weeks, 2.74 (1.95–3.54), *p* < 0.001], an older age [age <45, 2.09 (0.78–3.40), *p* = 0.002; 45 ≤ age <60, 2.25 (1.49–3.01), *p* < 0.001; age ≥60, 2.62 (1.31–3.94), *p* < 0.001], a larger basal body mass index (BMI) [20 < BMI < 25, 1.43 (0.98–1.88), *p <* 0.001; 25 ≤ BMI < 30, 2.49 (1.07–3.90), *p* < 0.001; BMI ≥ 30, 3.05 (1.69–4.42), *p <* 0.001], and a worse basal FMD [FMD < 4, 2.71 (0.92–4.49), *p* = 0.003; 4 ≤ FMD < 7, 2.63 (2.03–3.23), *p* < 0.001] were associated with larger improvements in FMD.

**Conclusion:** Continuous aerobic exercise, especially moderate-intensity and vigorous-intensity aerobic exercise, contributed to improving FMD. The effect of continuous aerobic exercise on improving FMD was associated with duration and participant’s characteristics. Specifically, a longer duration, an older age, a larger basal BMI, and a worse basal FMD contributed to more significant improvements in FMD.

**Systematic Review Registration**: [https://www.crd.york.ac.uk/PROSPERO/display_record.php?RecordID=341442], identifier [CRD42022341442].

## Introduction

The most common cause of mortality and morbidity worldwide is cardiovascular disease (CVD) (Townsend et al., 2015; Timmis et al., 2018; Kajikawa and Higashi, 2021), and studies have shown that CVD increases the incidence and mortality of other diseases (Clerkin et al., 2020), e.g., patients with underlying CVD may be at an increased risk for COVID-19 infection and mortality (Huang et al., 2020). Early evidence suggests that endothelial dysfunction, especially impaired endothelium-dependent vasodilation, is closely correlated with the emergence of various CVDs, such as hypertension, atherosclerosis, cardiac failure, and apoplexia (Lloyd-Jones et al., 2010; Tornero-Aguilera et al., 2022). Endothelial dysfunction is the initiating link of atherosclerosis (Thijssen et al., 2011), and early intervention in this link can effectively reverse the development of atherosclerosis (Thijssen et al., 2019). Flow-mediated dilation (FMD) describes the vasodilatory response to increasing shear stress in the brachial artery, which is currently used as a golden standard in evaluating endothelial function (Anderson and Mark, 1989). Several studies have shown that brachial artery FMD is a freestanding predictor of cardiovascular events (Gokce et al., 2002) and all-cause mortality (Xu et al., 2014a).

There is no doubt that absence of physical activity is one of the most significant CVD risk factors that can be modified (Haskell et al., 2007; Lee et al., 2012). The epidemiologic studies have shown that regular exercise, especially regular aerobic exercise, can promote cardiovascular function and reduce overall disease mortality (Bernardo et al., 2018; Wu et al., 2019). And even substantial evidence suggests that people who are physically active have a much higher percentage of survivors after a cardiovascular incident than those who are sedentary, and also describes the beneficial effects of physical activity on heart failure (Brandt et al., 2018). Previous studies have shown that aerobic exercise is superior to other types of exercise in improving vascular endothelial function (Kwon et al., 2011; O'Brien et al., 2020). Short-term aerobic exercise has been shown to significantly ameliorate the function of the endothelium of the brachial artery in male patients with chronic stable heart failure (Belardinelli et al., 2005). Meanwhile, a randomized controlled study found that 12 weeks of continuous aerobic exercise did not significantly improve endothelial function (Molmen-Hansen et al., 2012). And the relationship between aerobic exercise intensity and improvement in endothelial function is currently controversial. One study found that vigorous-intensity aerobic exercise significantly ameliorated endothelial function, whereas moderate-intensity aerobic exercise did not improve FMD (You et al., 2021). However, some studies show that low- or vigorous-intensity aerobic exercise does not improve FMD, a moderate-intensity aerobic exercise program can improve endothelial-dependent vasodilation (Goto et al., 2003; Goto et al., 2007). Therefore, the duration and the intensity of the continuous aerobic exercise are the key factors that affect the effects of intervene. Additionally, aerobic exercise affects vascular endothelial function differently based on the characteristics of the individual. However, some studies showed that aerobic exercise has no significant associations with FMD and baseline endothelial function in non-elderly healthy men and obese patients with type 2 diabetes (Wycherley et al., 2008; Swift et al., 2012; Shenouda et al., 2017).

Therefore, this systematic review and meta-analysis was conducted to investigate the effect of different durations and intensities of continuous aerobic exercise on the vascular endothelial function in people with different characteristics.

## Methods

### Design

According to PRIMA guidelines, this meta-analysis was conducted following preferred reporting items for systematic reviews and meta-analysis (PRISMA) (Page et al., 2021). The protocol for this systematic review has been registered on PROSPERO (CRD42022341442).

### Search strategy

For this systematic review and meta-analysis, we searched PubMed, Web of Science, and EBSCO electronic databases, through December 2021. The initial search consisted of the following MESH terms and keywords: aerobic exercise, flow-mediated dilation, and vascular endothelial function. We also hand-searched reference lists of all identified studies and, in addition, references of reviews and meta-analyses for any additional relevant studies that could be added to the relevant literature. Two authors (XT and YC) completed the process independently using a standardized form. If there was disagreement between the two authors, a third author (LY) would join the discussion until the three reach a consensus.

### Eligibility criteria

We included studies that satisfied the following criteria: eligible studies should 1) be randomized controlled trial (RCTs); 2) include both an intervention and control group with the only difference between them being the addition of continuous aerobic exercise in the intervention group; 3) use FMD as the outcome measure; and 4) the location of the FMD test was the brachial artery. In the analysis, non-English published articles, animal model publications, reviews, and conference articles were excluded.

### Data extraction

Two authors (XT and YC) independently performed the data extraction, mainly including: 1) characteristics of included studies (first author’s last name, year of study publication); 2) characteristics of continuous aerobic exercise (intensity, frequency, duration, session duration); 3) participant’s characteristics [*n*, gender, age, basal FMD, and basal body mass index (BMI)]; 4) treatment effects [mean and standard deviation (SD) values reflecting the change in FMD from baseline and to post-intervention in the continuous aerobic exercise and control groups. The corresponding authors of the original study were emailed when the results of the original study were incomplete.

### Methodological quality assessment

According to the Cochrane risk of bias criteria, we assessed the methodological quality of the studies included in this review (Higgins et al., 2011), and the quality assessment of eligible studies was based on factors including selection bias, performance bias, detection bias, attrition bias, reporting bias, and other biases. Independent assessments of methodological quality were conducted by two authors (XT and YC), and disagreements were resolved through discussion and consensus with a third author (LY).

### Statistical analysis

Each study’s mean and SD values reflecting the change in FMD from baseline to post-intervention have been extracted for pooling purposes. Data were pooled using random-effects models to obtain the weighted mean differences (WMDs) and 95% confidence intervals (CIs). When *I* ^2^ is < 50%, data were pooled using fixed effects models to obtain the WMD and 95% CIs; when *I* ^2^ is ≥ 50%, data were pooled using random effects models to obtain the WMD and 95% CIs (Li et al., 2022). If there was a high heterogeneity (*I* ^2^ > 60%), sensitivity analysis and subgroup analysis were used to interpret the results (Shamseer et al., 2015; Zhang et al., 2022; Zhen et al., 2022). In the subgroup analyses, we attempted to use durations of continuous aerobic exercise (<12 weeks, ≥12 weeks), intensities of continuous aerobic exercise (low-intensity, moderate-intensity, vigorous-intensity), participants’ age (young, age < 45; middle-aged, 45 ≤ age <60; elderly, age ≥60), basal BMI (normal weight, 20 < BMI < 25; overweight, 25 ≤ BMI < 30; obese, BMI ≥ 30) (Pi-Sunyer et al., 1998), and basal FMD (abnormal, FMD < 4; critical, 4 ≤ FMD < 7; normal, FMD ≥ 7) (Tanaka et al., 2018) to explore the impact on FMD. The analysis result, funnel plots, and forest plots were generated using RevMan 5.0 software. In terms of overall impact, *p* < 0.05 was considered statistically significant.

## Results

### Study selection

**FIGURE 1:**
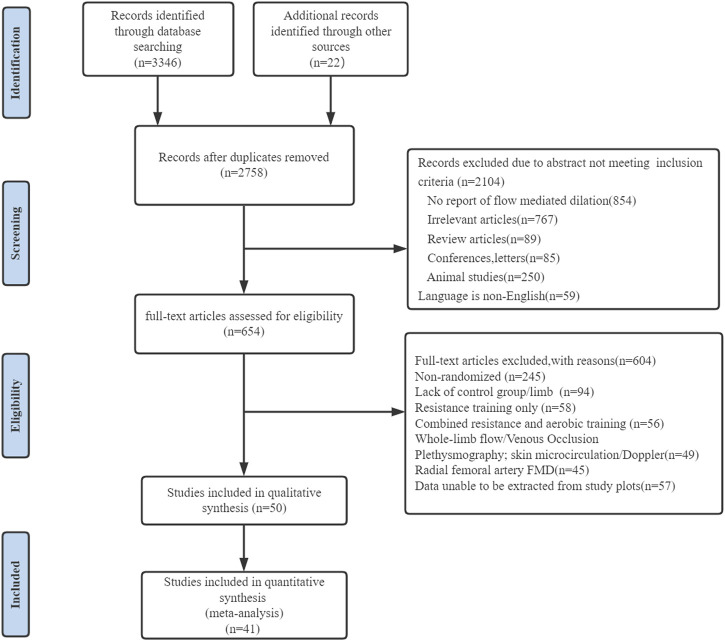
PRISMA flowchart of study selection.

Figure 1 showed the situation, preliminary results have been retrieved for 3,346 search records, and 22 records have been identified through other means. Following the exclusion of duplicate studies, 2,758 studies remained, and 2,104 studies were not eligible for inclusion through the title and abstract screening. Six hundred and four studies were excluded by reading the full text of 654 studies: 1) non-RCTs (*n* = 245); 2) no control group (*n* = 94); 3) the intervention was resistance training (*n* = 58); 4) a combination of treatments was used in the experimental group (*n* = 56); 5) no relevance was found in the outcomes (*n* = 94); 3); and 6) data could not be extracted (*n* = 57). Finally, 50 studies examining the effect of continuous aerobic exercise on FMD were considered eligible for systematic review, of which 41 studies were considered eligible for meta-analysis.

### Description of the included studies

The main characteristics of interventions and participants were presented in Table 1. There were 1959 participants enrolled in the 41 studies. Of the 41 studies, six studies involved diabetes participants (Wycherley et al., 2008; Kwon et al., 2011; Choi et al., 2012; Mitranun et al., 2014; Ghardashi et al., 2018; Boff et al., 2019), five studies involved healthy participants (Yoshizawa et al., 2010; Pierce et al., 2011; Shenouda et al., 2017; King and Pyke, 2020; Haynes et al., 2021), five studies involved high blood pressure participants (Westhoff et al., 2007; Westhoff et al., 2008; Molmen-Hansen et al., 2012; Beck et al., 2013; Boeno et al., 2020), four studies involved obese participants (Kelly et al., 2004; Meyer et al., 2006; Swift et al., 2012; Bhutani et al., 2013), four studies involved heart failure participants (Kobayashi et al., 2003; Belardinelli et al., 2005; Wisløff et al., 2007; Erbs et al., 2010), three studies involved metabolic syndrome participants (Lavrencic et al., 2000; Tjønna et al., 2008; Tjønna et al., 2011), three studies involved coronary artery disease participants (Sixt et al., 2008; Vona et al., 2009; Desch et al., 2010), two studies involved heart disease participants (Blumenthal et al., 2005; Braith et al., 2008), two studies involved hormonal diseases participants (Sprung et al., 2013; Jones et al., 2014), one study involved myocardial infarction participants (Vona et al., 2004), one study involved coronary heart disease (CHD) participants (Swift et al., 2012), one study involved peripheral arterial disease participants (McDermott et al., 2009), one study involved depression participants (Sherwood et al., 2016), one study involved kidney disease participants (Kirkman et al., 2019), one study involved non-alcoholic fatty liver disease (NAFLD) participants (Pugh et al., 2014), and one study involved high cardiovascular risk participants (Jo et al., 2020). In studies examining the effect of continuous aerobic exercise on endothelial function in diseased participants, drug use did not change throughout the intervention period. In addition, gender was not a factor in about 80% of studies, with only five studies targeting women (Yoshizawa et al., 2010; Kwon et al., 2011; Choi et al., 2012; Sprung et al., 2013; Jo et al., 2020) and four studies targeting men (Lavrencic et al., 2000; Jones et al., 2014; Shenouda et al., 2017; King and Pyke, 2020).

**TABLE 1:** Characteristics of included study participants.

Studies	Participants				Exercise intervention				Results on FMD
	Gender (M/F)	Age y)	Population	Type	Intensity	Duration	Frequency	Times	
Beck et al. (2013)	IG: 9/4	IG: 20.1 ± 1.1	Prehypertension	Walking/running	65%–85% HR_max_	8 weeks	3 days/week	60 min/day	Increase
	CG: 9/6	CG: 21.6 ± 2.7							
Belardinelli et al. (2005)	IG: 30/0	IG: 55.9 ± 15	CHF	Cycling	60% VO_2max_	8 weeks	3 days/week	60 min/day	Increase
	CG: 29/0	CG: 58 ± 12							
Bhutani et al. (2013)	IG: 23/1	IG: 42 ± 2	Obese adults	Cycling	60%–75% HR_max_	12 weeks	3 days/week	40 min/day	No change
	CG: 15/10	CG: 49 ± 2							
Boeno et al. (2020)	IG: 8/7	IG: 45.8 ± 6.8	Hypertension	Walking/running	60%–80% HRR	12 weeks	3 days/week	55–60 min/day	Increase
	CG: 5/7	CG: 44.3 ± 8.3							
Boff et al. (2019)	IG: 5/4	IG: 23.7 ± 5.8	T1DM	Cycling	50%–65% HR_max_	8 weeks	3 days/week	40 min/day	No change
	CG: 4/5	CG: 20.8 ± 2.6							
Braith et al. (2008)	IG: 9	IG: 54.4 ± 13.1	Heart transplant recipients	Walking	RPE:12-14	12 weeks	3 days/week	35–40 min/day	No change
	CG:7	CG: 54.3 ± 9.5							
Choi et al. (2012)	IG: 38	IG: 53.8 ± 7.2	T2DM	Walking	65%–85% HR_max_	8 weeks	3 days/week	60 min/day	Increase
	CG: 37	CG: 55.0 ± 6.0							
Desch et al. (2010)	IG: 11/3	IG: 62.3 ± 6.2	CAD	Cycling	75% HR_max_	24 weeks	3 days/week	30 min/day	Increase
	CG: 8/4	CG: 62.3 ± 6.5							
Erbs et al. (2010)	IG: 18	IG: 60 ± 11	CHF	Cycling	60% VO_2max_	12 weeks	7 days/week	20–30 min/day	Increase
	CG: 19	CG: 62 ± 10							
Ghardashi et al. (2018)	IG: 7/10	IG: 53.1 ± 4.8	T2DM	Cycling	70% HR_max_	12 weeks	3 days/week	42 min/day	Increase
	CG: 9/8	CG: 54.2±5.61							
Haynes et al. (2021)	IG: 3/14	IG: 61.9 ± 5.4	Healthy adults	Walking	55%–65% HR_max_	24 weeks	3 days/week	20–30 min/day	No change
	CG: 4/12	CG: 61.8 ± 7.3							
Haynes et al. (2021)	IG: 3/15	IG: 62.2 ± 7.4	Healthy adults	Walking	55%–65% HR_max_	24 weeks	3 days/week	20–30 min/day	Increase
	CG: 4/12	CG: 61.8 ± 7.3							
Blumenthal et al. (2005)	IG: 32/10	IG: 62 ± 10.5	IHD	Walking	50–85%HRR	16 weeks	3 days/week	35 min/day	No change
	CG: 31/17	CG: 63 ± 9							
Jo et al. (2020)	IG: 0/22	IG: 61.8 ± 10.1	PW + high cardiovascular risk	Running	42–82%HRR	12 weeks	3 days/week	40 min/day	Increase
	CG: 0/21	CG: 62 ± 13.9							
Jo et al. (2020)	IG: 0/22	IG: 57.3 ± 8.4	PW + high cardiovascular risk	Walking/jogging	60%–80% HR_max_	12 weeks	3 days/week	40 min/day	Increase
	CG: 0/21	CG: 62.5±13.9							
Jones et al. (2014)	IG: 25/0	IG: 58 ± 5	PCA	Walking	55%–100% HR_max_	24 weeks	5 days/week	30–45 min/day	Increase
	CG: 25/0	CG: 61 ± 5							
Kelly et al. (2004)	IG: 5/5	IG: 11 ± 0.6	Overweight children	Cycling	50%–80% HR_max_	8 weeks	4 days/week	20–30 min/day	Increase
	CG: 4/6	CG: 11 ± 0.7							
King and Pyke (2020)	IG: 16/0	IG: 22 ± 3	Healthy adults	Cycling	80% HRR	4 weeks	3 days/week	30 min/day	Increase
	CG: 12/0	CG: 21 ± 2							
Kirkman et al. (2019)	IG: 5/10	IG: 55 ± 13	CKD	Cycling	60%–85% HRR	12 weeks	3 days/week	45 min/day	Increase
	CG: 4/12	CG: 62 ± 9							
Kobayashi et al. (2003)	IG: 12/2	IG: 55 ± 2	CHF	Cycling	Borg: 13	12 weeks	2–3 days/week	30 min/day	No change
	CG: 8/6	CG: 62 ± 2							
Kwon et al. (2011)	IG: 0/13	IG: 55.5 ± 8.6	T2DM	Not clear	3.6–6.0 METs	12 weeks	5 days/week	60 min/day	Increase
	CG: 0/15	CG: 58.9 ± 5.7							
Lavrencic et al. (2000)	IG: 14	IG: 53 ± 5	MS	Cycling	80% HR_max_	12 weeks	3 days/week	50 min/day	Increase
	CG: 15	CG: 51 ± 7							
McDermott et al. (2009)	IG: 24/27	IG: 71.7 ± 8.7	PAD	Walking	RPE: 12-14	24 weeks	3 days/week	15–40 min/day	Increase
	CG: 25/28	CG: 68.5±11.9							
Meyer et al. (2006)	IG: 33	IG: 14.2 ± 1.9	Obese children	Not clear	Not clear	24 weeks	3 days/week	60–90 min/day	Increase
	CG: 34	CG: 14.7 ± 2.2							
Mitranun et al. (2014)	IG: 5/9	IG: 61.7 ± 2.7	T2DM	Walking	60%–65% VO_2max_	12 weeks	3 days/week	40 min/day	Increase
	CG: 5/10	CG: 60.9 ± 2.4							
Molmen-Hansen et al. (2012)	IG: 16/12	IG: 53.6 ± 6.5	Hypertension	Walking/running	60% VO_2max_	12 weeks	3 days/week	47 min/day	No change
	CG: 17/12	CG: 51.3 ± 9.2							
Tjønna et al. (2011)	IG: 8	Not clear	MS	Walking/running	70% HR_max_	16 weeks	3 days/week	47 min/day	Increase
	CG: 9								
Pierce et al. (2011)	IG: 11/15	IG: 63 ± 1	Healthy adults	Not clear	70%–75% HR_max_	8 weeks	6 days/week	50 min/day	Increase
	CG: 10	CG: 60 ± 1							
Pugh et al. (2014)	IG: 22/12	IG: 48 ± 15.3	NAFLD	Cycling	30%–60% HRR	16 weeks	3 days/week	30–45 min/day	Increase
	CG: 8/12	CG: 47 ± 13.1							
Belardinelli et al. (2006)	IG: 15	IG: 55.1 ± 14	CHF + Implantable cardioverter defibrillator	Cycling	60% VO_2max_	8 weeks	3 days/week	60 min/day	Increase
	CG: 12	CG: 55.1 ± 14							
Belardinelli et al. (2006)	IG: 15	IG: 53.1 ± 15	CHF + Implantable cardioverter defibrillator + cardiac resynchronization therapy	Cycling	60% VO_2max_	8 weeks	3 days/week	60 min/day	Increase
	CG: 10	CG: 53.1 ± 15							
Shenouda et al. (2017)	IG: 10	IG: 28 ± 9	Healthy adults	Cycling	55% VO_2max_	6 weeks	3 days/week	50 min/day	Increase
	CG: 6	CG: 26 ± 8							
Shenouda et al. (2017)	IG: 10	IG: 28 ± 9	Healthy adults	Cycling	55% VO_2max_	12 weeks	3 days/week	50 min/day	No change
	CG: 6	CG: 26 ± 8							
Sherwood et al. (2016)	IG: 12/39	IG: 51.1 ± 7.0	MDD	Walking/jogging	70%–85% HRR	24 weeks	3 days/week	30 min/day	Increase
	CG: 11/38	CG: 51.2 ± 7.8							
Sherwood et al. (2016)	IG: 14/39	IG: 52.8 ± 7.9	MDD	Not clear	70%–85% HRR	24 weeks	3 days/week	30 min/day	Increase
	CG: 4/12	CG: 51.2 ± 7.8							
Sixt et al. (2008)	IG: 10/3	IG: 64 ± 6	DM + CHD	Cycling	70% HR_max_	4 weeks	6 days/week	15 min/day	Increase
	CG: 7/3	CG: 64 ± 6							
Sprung et al. (2013)	IG: 0/10	IG: 28 ± 9.8	PCOS	Not clear	30%–60% HRR	16 weeks	5 days/week	30 min/day	Increase
	CG: 0/7	CG: 28 ± 9.8							
Swift et al. (2012)	IG: 0/68	IG: 57.4 ± 5.8	Hypertension + PW	Cycling	50% VO_2max_	24 weeks	Not clear	Not clear	Increase
	CG: 0/23	CG: 56.8 ± 5.4							
Swift et al. (2012)	IG: 0/32	IG: 55.9 ± 6.0	Hypertension + PW	Cycling	50% VO_2max_	24 weeks	Not clear	Not clear	Increase
	CG: 0/23	CG: 56.8 ± 5.4							
Swift et al. (2012)	IG: 0/32	IG: 56.3 ± 6.8	Hypertension + PW	Cycling	50% VO_2max_	24 weeks	Not clear	Not clear	Increase
	CG: 0/23	CG: 56.8 ± 5.4							
Tjønna et al. (2008)	IG: 4/4	IG:52 ± 10.6	MS	Walking/running	70% VO_2max_	16 weeks	3 days/week	47 min/day	Increase
	CG: 5/4	CG:49.6 ± 9							
Vona et al. (2004)	IG: 21/7	IG: > 70	MI	Cycling	75% HR_max_	12 weeks	3 days/week	40 min/day	Increase
	CG: 19/5	CG: > 70							
Vona et al. (2009)	IG: 39/13	IG: 56 ± 6	MI	Cycling	75% HR_max_	4 weeks	4 days/week	60 min/day	Increase
	CG: 37/13	CG: 58 ± 7							
Westhoff et al. (2007)	IG: 14/13	IG: 67.2 ± 4.8	Healthy adults	Walking	Not clear	12 weeks	3 days/week	30 min/day	Increase
	CG: 14/13	CG: 68.9 ± 5.2							
Westhoff et al. (2008)	IG: 5/7	IG: 66.1 ± 4	Hypertension	Upper-limb cycling	Not clear	12 weeks	3 days/week	30 min/day	Increase
	CG: 6/6	CG: 68.4 ± 9.7							
Wisløff et al. (2007)	IG: 7/2	IG: 74.4 ± 12	CHF	Walking	70%–75% HR_max_	12 weeks	3 days/week	47 min/day	Increase
	CG: 6/3	CG: 75.5 ± 13							
Wycherley et al. (2008)	IG: 6/7	IG: 51.7 ± 2.4	T2DM	Walking/jogging	60%–80% HR_max_	12 weeks	4–5 days/week	25–60 min/day	Increase
	CG: 10/6	CG: 53 ± 1.8							
Yoshizawa et al. (2010)	IG: 0/10	IG: 57 ± 1	PW	Walking/cycling	60%–75% HR_max_	8 weeks	3–5 days/week	20–30 min/day	Increase
	CG: 0/10	CG: 58 ± 1							

Abbreviation: M, male; F, female; y, year; IG, intervention groups; CG, control groups; VO_2max_, maximal oxygen consumption; HR_max_, maximum heart rate; HRR, heart rate reserve; METs, metabolic equivalents; RPE, rating of perceived exertion; CAD, coronary artery disease; MS, metabolic syndrome; NAFLD, non-alcoholic steatohepatitis; IHD, ischemic heart disease; PW, postmenopausal women; PCA, prostate cancer; CKD, chronic kidney disease; MDD, major depressive disorder; PAD, peripheral arterial disease; CHF, chronic heart failure; T1DM, type 1 diabetes mellitus; T2DM, type 2 diabetes mellitus; PCOS, polycystic ovarian syndrome; MI, myocardial infarction; CHD, coronary artery heart disease; DM, diabetic mellitus; FMD, flow-mediated dilation.

The overall duration varied from 4 weeks to 24 weeks. As stated in the position statement regarding physical activity and training intensity (Norton et al., 2010), following are the modifications we made to the classification of continuous aerobic exercise intensity according to the included research situation: 1.6 < metabolic equivalents (METs) < 3, 20% < maximal oxygen uptake (VO_2max_) < 40%, 40% < maximal heart rate (HR_max_) < 55%, 20% < heart rate reserve (HRR) < 40%, or 8 < rating of perceived exertion (RPE) < 10 were determined as low-intensity; 3 < METs < 6, 40% < VO_2max_ < 60%, 55% < HR_max_ < 70%, 40% < HRR < 60%, or 11 < RPE < 13 were determined as moderate-intensity; 6 < METs < 9, 60% < VO_2max_ < 85%, 70% < HR_max_ < 90%, 60% < HRR < 85%, or 14 < RPE < 16 were determined as vigorous-intensity.

### Risk of bias

**FIGURE 2:**
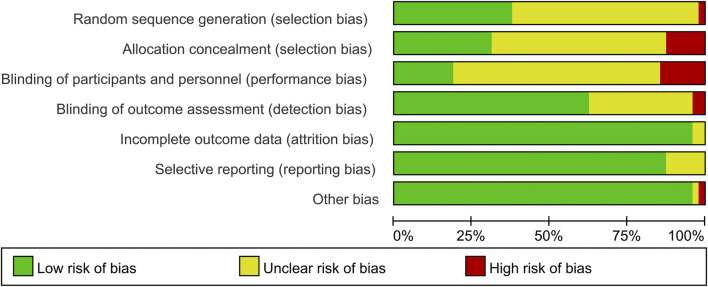
Risk of bias summary.

**FIGURE 3:**
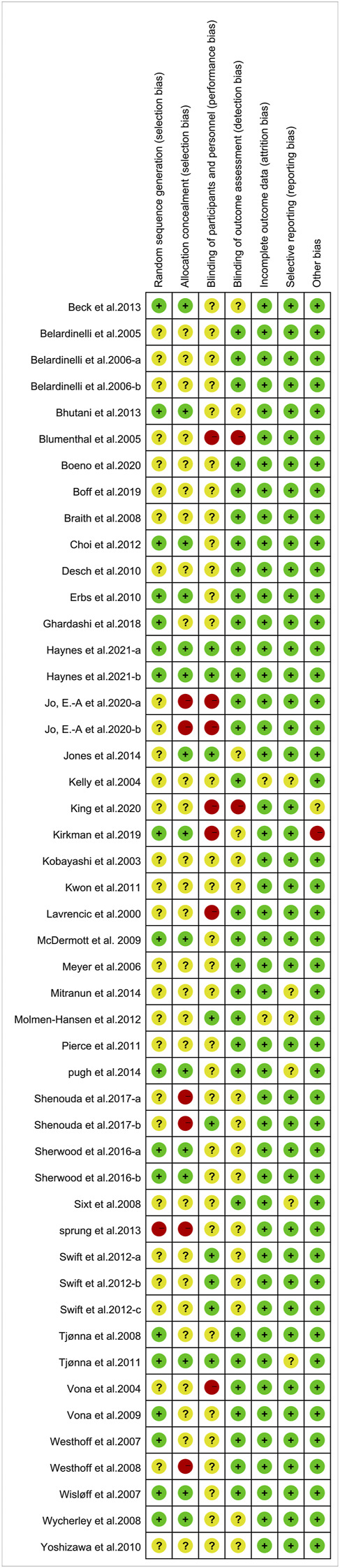
Risk of bias graph.

**FIGURE 4:**
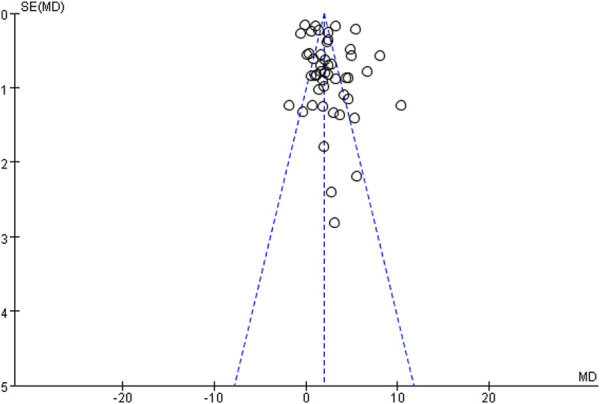
Funnel plot.

Evaluation of the methodological quality of the included literature was conducted using the Cochrane risk assessment tool, mainly from selection bias, performance bias, detection bias, attrition bias, reporting bias and other biases. Quality was scored according to three levels (low risk, high risk and unclear). The quality of the included literature is divided into three levels from high to low: high quality, medium quality and low quality (Figures 2, 3). Publication bias was assessed visually by inspecting the funnel plot (Figure 4).

### Meta-analysis results

#### The effect of continuous aerobic exercise on FMD

**FIGURE 5:**
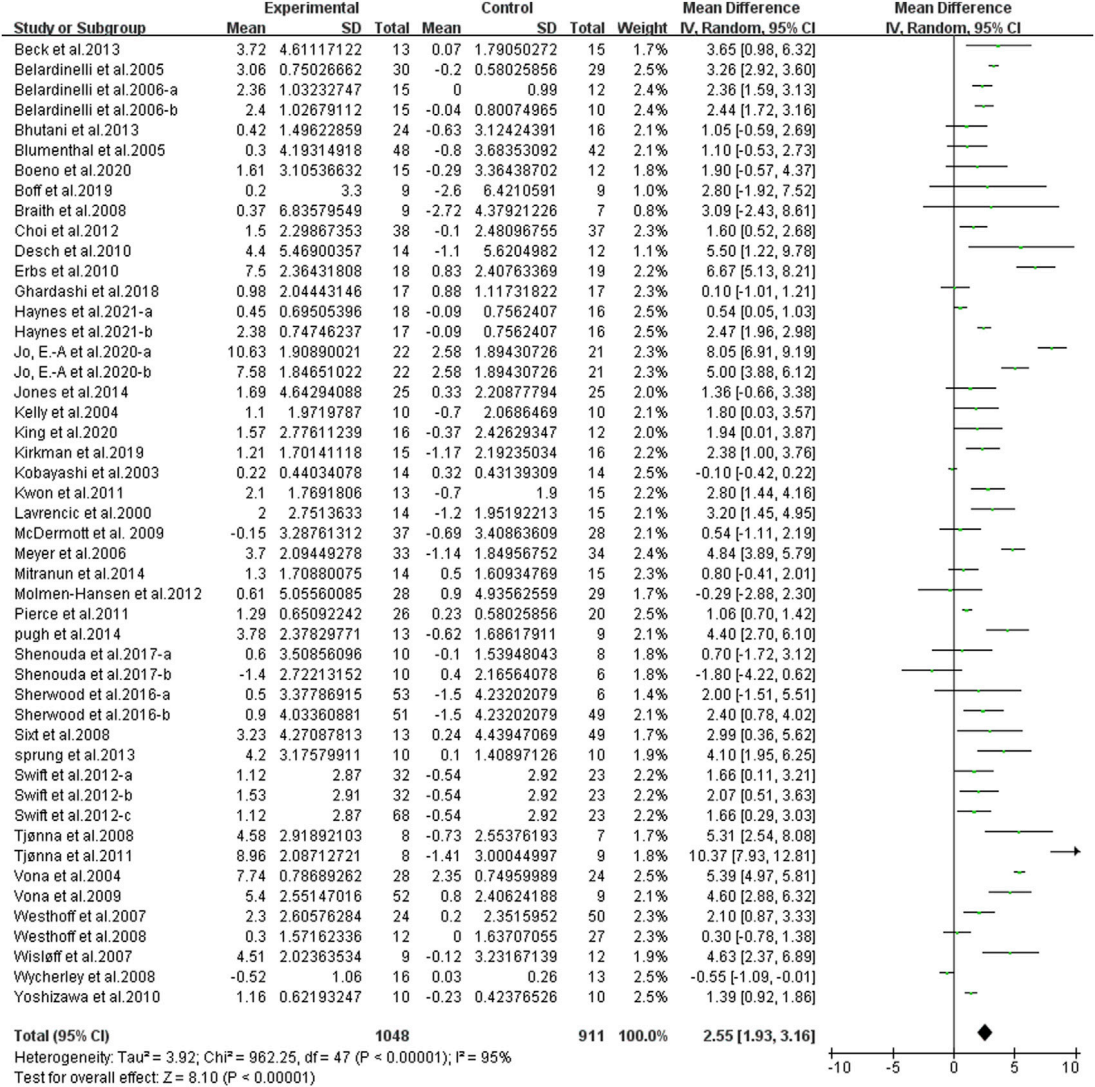
Meta-analysis results of the effect of continuous aerobic exercise on FMD. The pooled estimates were obtained from random effects analysis. Diamonds indicated the effect size of each study summarized as WMD. The size of the shaded squares was proportional to the percentage weight of each study. Horizontal lines represented the 95% CI and the vertical line represented the overall effect.

We found that continuous aerobic exercise had a significant effect on increasing FMD when compared to the control group [WMD, 2.55 (95% CI, 1.93–3.16), *p* < 0.001, Figure 5], while there was a significant heterogeneity (*I* ^2^ = 95%). Hence, a sensitivity analysis in which 1 study was removed at a time, was performed to evaluate the stability of the results. Sensitivity analysis results were shown in Supplementary Figure S1, the pooled effect changed slightly by removing each study, which confirmed the stability of our results. Therefore, we used subgroup analyses to interpret the results.

#### Subgroup analysis

**FIGURE 6:**
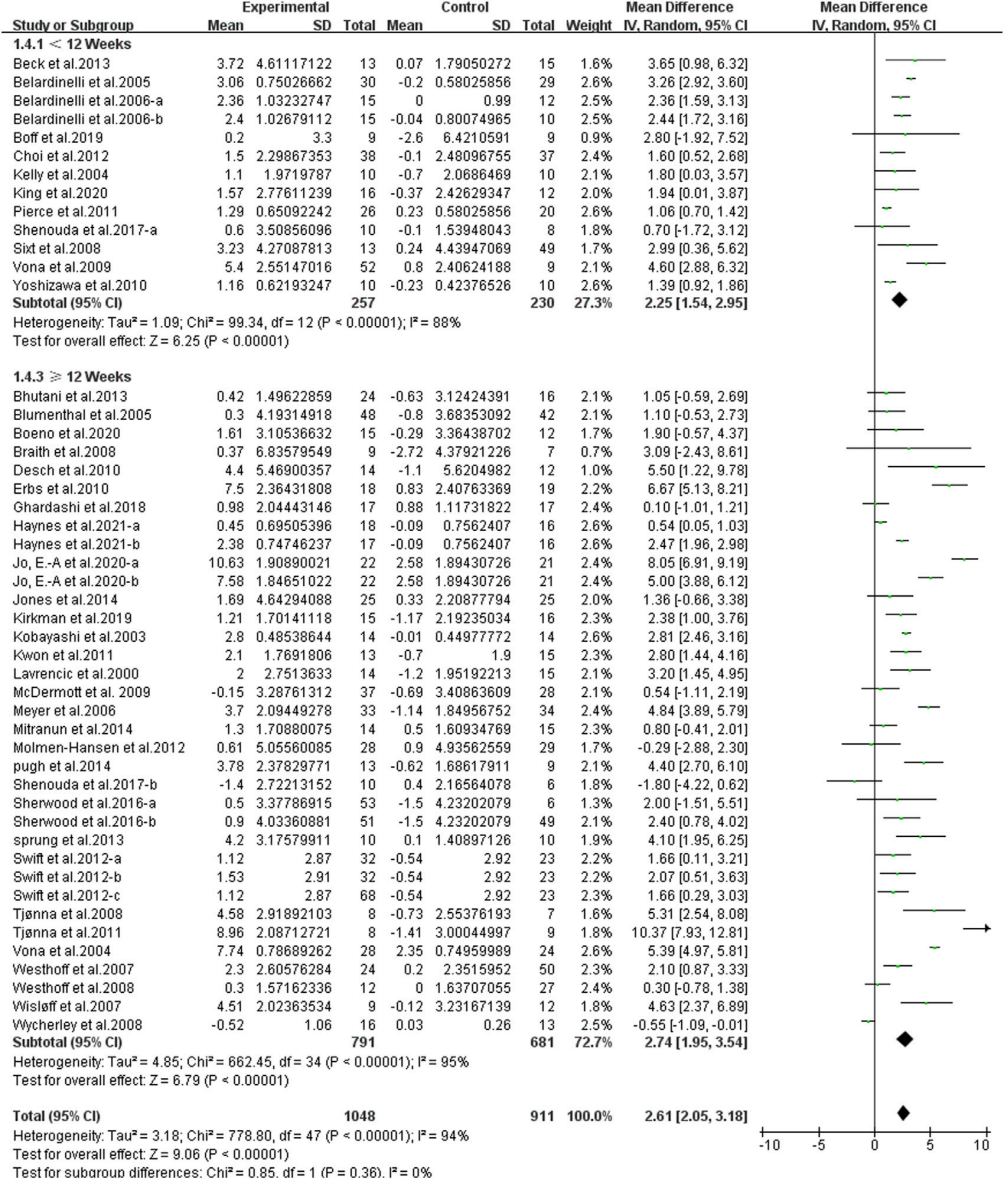
Meta-analysis results of the effect of different durations of continuous aerobic exercise on FMD. The pooled estimates were obtained from random effects analysis. Diamonds indicated the size of the effect of each study summarized as WMD. The size of the shaded square was proportional to the percentage weight of each study. Horizontal lines represented the 95% CI and the vertical dashed line represented the overall effect.

Different results were shown when considering duration of intervention (Figure 6). The subgroup analysis indicated that a longer duration was associated with larger improvements in FMD [< 12 weeks, WMD, 2.25 (95%CI, 1.54–2.95), *p* < 0.001, *I* ^2^ = 88%; ≥ 12 weeks, WMD, 2.74 (95%CI, 1.95–3.54), *p* < 0.001, *I* ^2^ = 95%].

**FIGURE 7:**
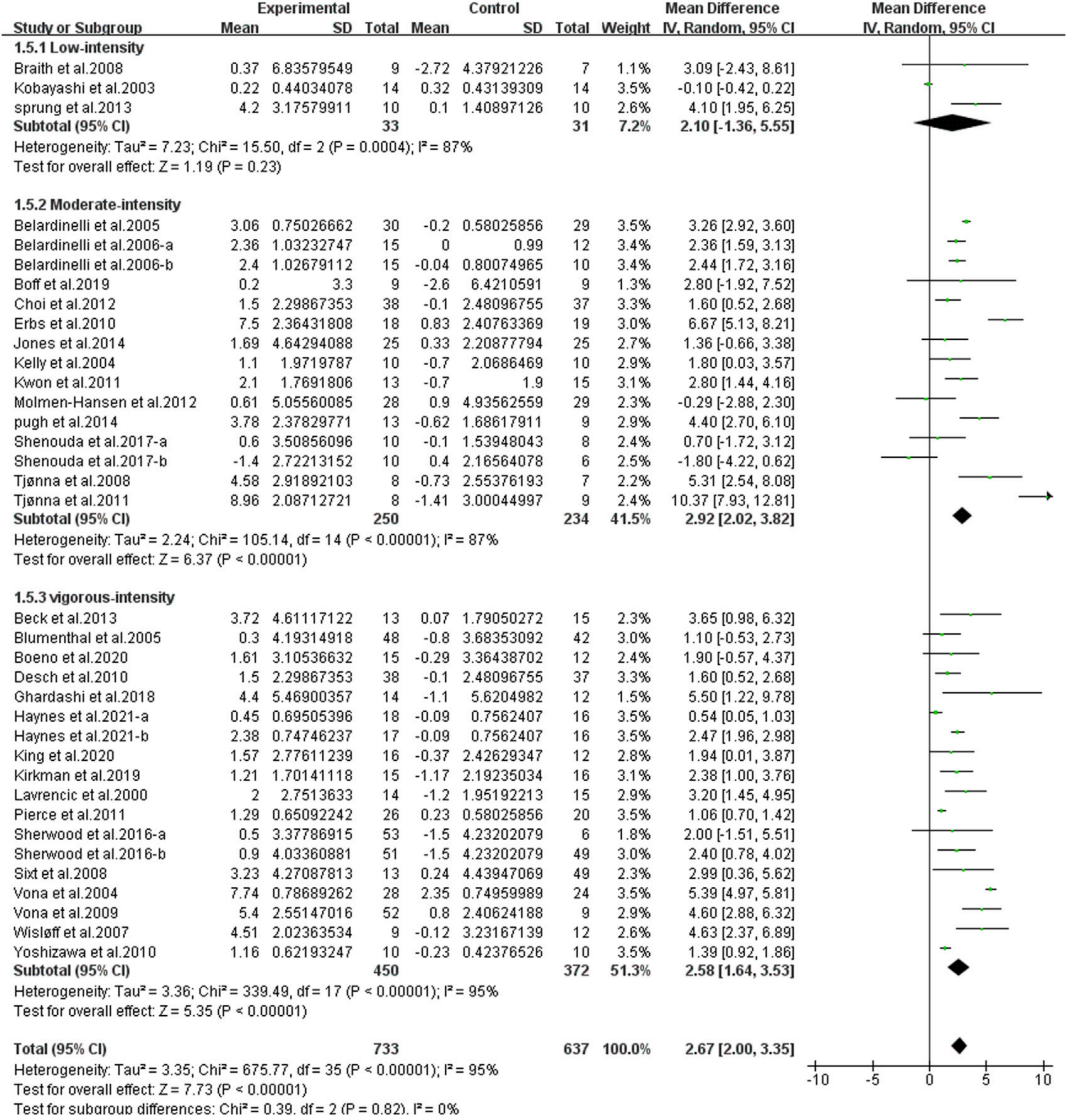
Meta-analysis results of the effect of different intensities of continuous aerobic exercise on FMD. The pooled estimates were obtained from random effects analysis. Diamonds indicated the size of the effect of each study summarized as WMD. The size of the shaded square was proportional to the percentage weight of each study. Horizontal lines represented the 95% CI and the vertical dashed line represented the overall effect.

Different results were shown when considering exercise intensity (Figure 7). Specifically, compared with the control group, moderate-intensity exercise [WMD, 2.92 (95%CI, 2.02–3.82), *p* < 0.001, *I* ^2^ = 87%] and vigorous-intensity exercise [WMD, 2.58 (95%CI, 1.64–3.53), *p* < 0.001, *I* ^2^ = 95%] significantly increased FMD, while low-intensity exercise had no significant effect on FMD [WMD, 2.10 (95%CI, −1.36–5.55), *p* = 0.23, *I* ^2^ = 87%].

**FIGURE 8:**
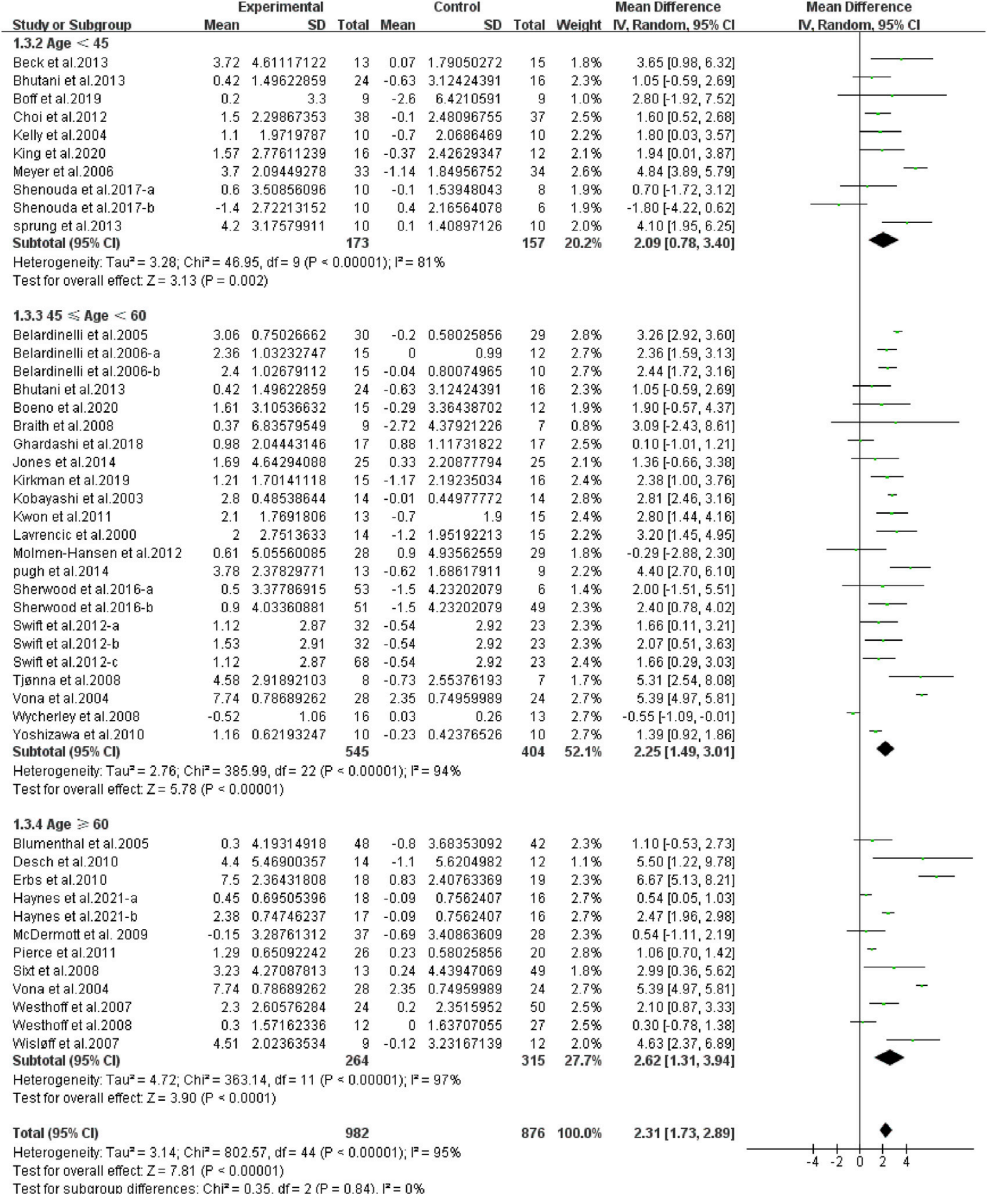
Meta-analysis results of the effect of continuous aerobic exercise on FMD in young, middle-aged, or elderly people. The pooled estimates were obtained from random effects analysis. Diamonds indicated the size of the effect of each study summarized as WMD. The size of the shaded square was proportional to the percentage weight of each study. Horizontal lines represented the 95% CI and the vertical dashed line represented the overall effect.

**FIGURE 9:**
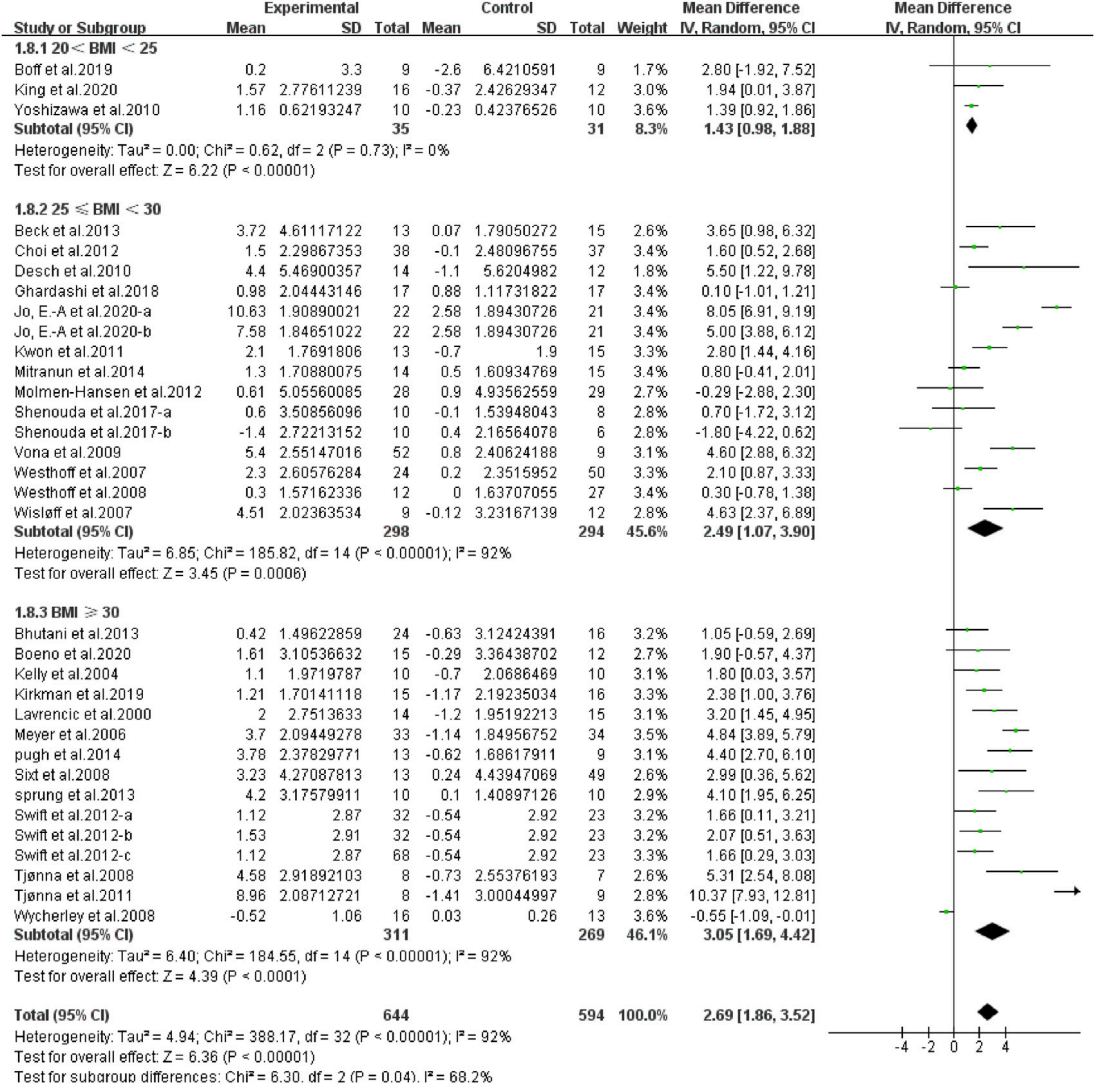
Meta-analysis results of the effect of continuous aerobic exercise on FMD in normal weight, overweight, or obese people. The pooled estimates were obtained from random effects analysis. Diamonds indicated the size of the effect of each study summarized as WMD. The size of the shaded square was proportional to the percentage weight of each study. Horizontal lines represented the 95% CI and the vertical dashed line represented the overall effect.

**FIGURE 10:**
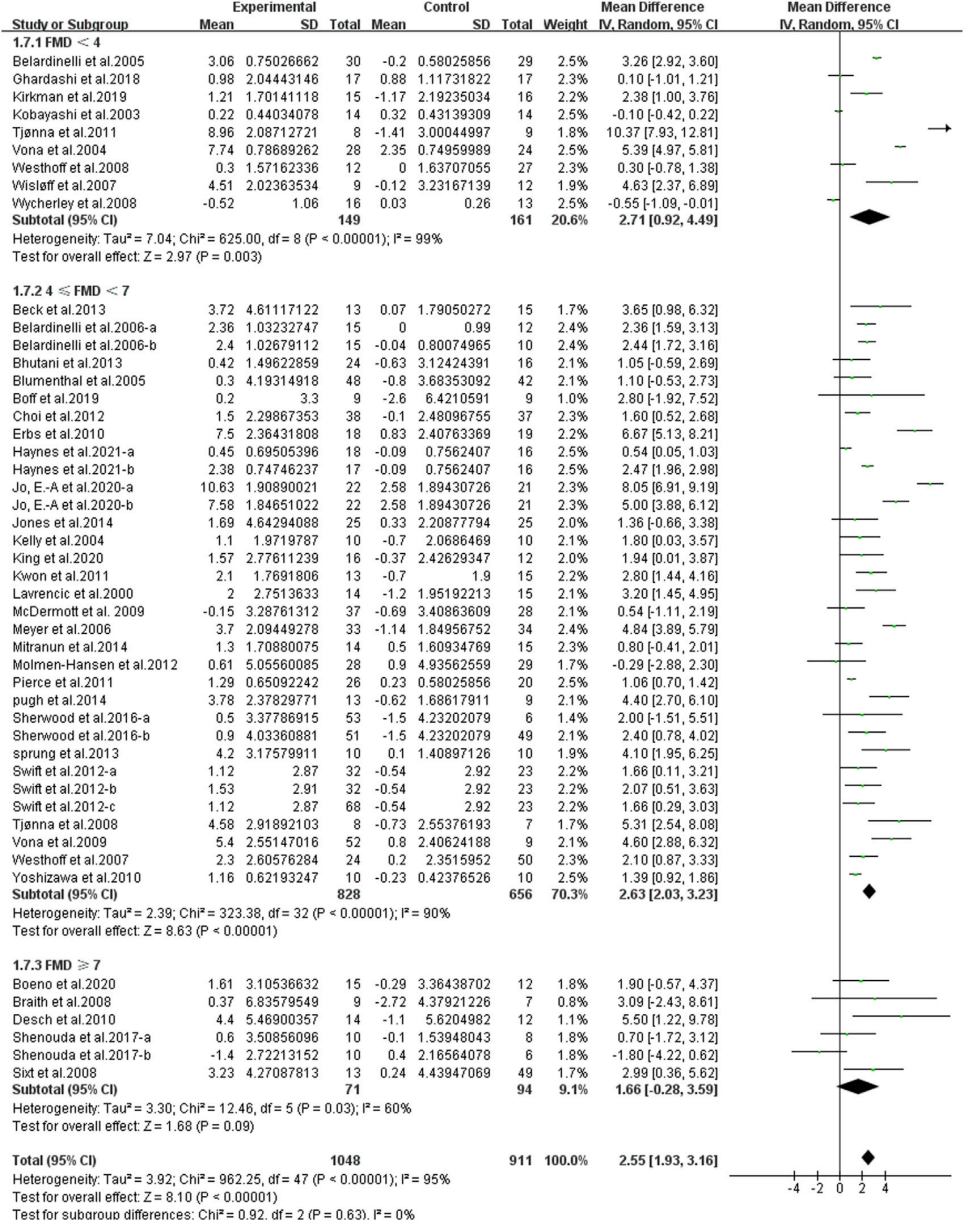
Meta-analysis results of the effect of continuous aerobic exercise on FMD in people with normal, critical, or abnormal basal FMD. The pooled estimates were obtained from random effects analysis. Diamonds indicated the size of the effect of each study summarized as WMD. The size of the shaded square was proportional to the percentage weight of each study. Horizontal lines represented the 95% CI and the vertical dashed line represented the overall effect.

Different results were shown when considering participants’ characteristics. The subgroup analysis indicated that an older age [young, age < 45, WMD, 2.09 (95%CI, 0.78–3.40), *p* = 0.002, *I* ^2^ = 81%; middle-aged, 45 ≤ age <60, WMD, 2.25 (95%CI, 1.49–3.01), *p* < 0.001, *I* ^2^ = 94%; elderly, age ≥60, WMD, 2.62 (95%CI, 1.31–3.94), *p* < 0.001, *I* ^2^ = 97%, Figure 8], a larger basal BMI [normal weight, 20 < BMI < 25, WMD, 1.43 (95%CI, 0.98–1.88), *p* < 0.001, *I* ^2^ = 0%; overweight, 25 ≤ BMI < 30, WMD, 2.49 (95%CI, 1.07–3.90), *p* < 0.001, *I* ^2^ = 92%; obese, BMI ≥ 30, WMD, 3.05 (95%CI, 1.69–4.42), *p* < 0.001, *I* ^2^ = 92%, Figure 9], and a worse basal FMD [abnormal, FMD < 4, WMD, 2.71 (95%CI, 0.92–4.49), *p* = 0.003, *I* ^2^ = 99%; critical, 4 ≤ FMD < 7, WMD, 2.63 (95%CI, 2.03–3.23), *p* < 0.001, *I* ^2^ = 90%, Figure 10] were associated with larger improvements in FMD. However, continuous aerobic exercise had no significant effect on FMD in people with normal basal FMD [WMD, 1.66 (95%CI, −0.28–3.59), *p* = 0.09, *I* ^2^ = 60%, Figure 10].

## Discussion

### The effect of continuous aerobic exercise on endothelial function

This systematic review and meta-analysis indicated that continuous aerobic exercise significantly improved endothelial function, as revealed by increased FMD, which was consistent with the results of You et al. (2021), showing that aerobic exercise improved endothelial function. Our study showed that continuous aerobic exercise contributed to an overall improvement in the FMD by 2.55 (WMD), which has a clinical importance for public health. The mechanism of continuous aerobic exercise improving vascular endothelial function had not been fully revealed, the following potential mechanisms might explain the beneficial effects of continuous aerobic exercise on vascular endothelial function.

First, flow shear stress (FSS), the frictional force exerted by blood on the vessel wall, is considered a regulator of the vascular endothelium (Girard and Nerem, 1995). Numerous studies have shown that aerobic exercise increase blood flow shear stress (Goto et al., 2003; Tinken et al., 2010) that can directly induce increased NO synthesis and release from vascular endothelium (Green et al., 2004; Thijssen et al., 2010), increasing NO bioavailability (Taddei et al., 2000) and secondary enhancement of endothelial nitric oxide synthase (eNOS) expression (Hambrecht et al., 2003; Newcomer et al., 2011). Second, it has been suggested that inflammation plays a central role in endothelial dysfunction (a critical step in the progression of CVD) (Rathod et al., 2017), aerobic exercise can reduce plasma biomarkers of low-grade inflammation [highly sensitive C-reactive protein (hs-CRP), serum amyloid A (SAA), soluble intercellular adhesion molecule-1 (sICAM-1), interleukin-6 (IL-6), interleukin-8 (IL-8), and tumor necrosis factor-α (TNF-α)], endothelial dysfunction biomarkers [soluble vascular adhesion molecule-1 (sVCAM-1) and soluble E-selectin (sE-selectin)] (Vandercappellen et al., 2022), levels of l-arginine, and ratios of l-arginine to citrulline, citrulline to ornithine, and l-arginine to ornithine (Tsukiyama et al., 2017). Third, oxidative stress is one of the risk factors for vascular endothelial disorders (Yilmaz et al., 2020). Aerobic exercise can modulate oxidative stress, increase the expression of antioxidant enzymes, and regulate reactive oxygen species (ROS) in mitochondria, and ROS induces nuclear transcriptional coactivators PPAR-gamma co-activator-1 alpha/beta (PGC1α/β) and activates several nuclear transcription factors, including peroxisome proliferator-activated receptor gamma (PPARγ) and its targets superoxide dismutase (SOD) 1, SOD2, glutathione peroxidase (GPx) 1, and catalase (CAT) (St-Pierre et al., 2006). Fourth, studies have shown that regular aerobic exercise can normalize the basal overactivity of the sympathetic nervous system (Antunes-Correa et al., 2010), muscle sympathetic nerve activity (MSNA) is a method that records sympathetic nerve passage, which is reduced by aerobic exercise (Notarius et al., 2015). NO also regulates vascular endothelial function by interacting with the autonomic nervous system (Gamboa et al., 2012). Finally, aerobic exercise can increase endothelial progenitor cell (EPC) number and differentiation capacity (Landers-Ramos et al., 2019), as well as vascular endothelial growth factor (VEGF) and insulin growth factor-1 (IGF-1) (Vonderwalde and Kovacs-Litman, 2018), which can contribute to vascular regeneration and angiogenesis, and aerobic exercise can promote the secretion of active protective factors, such as endothelin (ET), prostacyclin I2 (PGI2), angiotensin II (Ang II), arginase, and other biologically active molecules (Beck et al., 2013), which have a positive effect on endothelial function. It may be possible to use endothelial progenitor cells as a surrogate biologic marker for cardiovascular function and cumulative morbidity (Hill et al., 2003; Koutroumpi et al., 2012).

### Subgroup analysis

In the studies we included, continuous aerobic exercise significantly improved vascular endothelial function, while there was considerable heterogeneity between groups. Therefore, we used subgroup analysis to interpret the results. First, the effect of aerobic exercise is closely related to the duration and intensity of the intervention, so we performed subgroup analyses about intervention duration and exercise intensity. Our results showed that the duration of ≥ 12 weeks and < 12 weeks both improved the vascular endothelial function significantly, and ≥ 12 weeks of continuous aerobic exercise was more effective in improving FMD than < 12 weeks. Previous studies have found that short-term training can increase the bioavailability of eNOS and NO, thereby improving vascular endothelial function (Sessa et al., 1994; Sun et al., 1994; Kingwell et al., 1997). However, some studies have found that long-term training can expand the diameter of arteries (Leon and Bloor, 1968; Wyatt and Mitchell, 1978; Lash and Bohlen, 1992), the better effect of ≥ 12 weeks may be due to structural changes in the vessel caused by long-term repetitive training, a phenomenon sometimes referred to as “arterial remodeling’’ (Prior et al., 2003). Time-course studies have shown that training firstly induces functional changes, followed by structural remodeling that restores function to baseline levels (Laughlin, 1995). While some evidence suggests that diameter may not be significantly enlarged in all situation at rest, this may be due to compensatory increases in vasoconstrictor and relaxation tone to regulate blood pressure and vascular resistance, which can increase the flow shear stress, thereby improving the vascular endothelial function (Green et al., 2012). Aerobic exercise improves vascular endothelium, and there appears to be an optimal training intensity. In our study, moderate-intensity exercise and vigorous-intensity exercise significantly increased FMD, and moderate-intensity exercise having a greater effect than vigorous-intensity exercise, while low-intensity exercise had no significant effect on FMD, which was in line with the report by Goto et al. (2003), showing that low-intensity aerobic exercise has no effect on vascular endothelial function. In this context, low-intensity aerobic exercise may not provide the optimal shear stress stimulus with the potential to improve endothelial function (Green et al., 2004), whereas moderate-intensity and high-intensity aerobic exercise enhances endothelial-dependent vasodilation in humans by increasing NO production, blood flow, blood flow speed, and shear stress, and reducing ROS production (Goto et al., 2003; Harrison et al., 2006; Tinken et al., 2010; Ramos et al., 2015; Green et al., 2017). Therefore, we speculated that there may be a dose-response relationship between the duration of continuous aerobic exercise and FMD, and this relationship depends not only on duration but also on exercise intensity. Discovering the optimal effect of exercise duration and optimal intensity threshold may help to design more effective and safe clinical prescriptions for patients with endothelial dysfunction.

Studies have previously shown that increasing FMD is positively related to reducing the risk of CVD, and that every 1% increase in FMD in the brachial artery decreases risk by 13% (Inaba et al., 2010; Ras et al., 2013; Xu et al., 2014b; Matsuzawa et al., 2015; Thijssen et al., 2019). Therefore, the changes of CVD risk factors are closely related to the improvement of vascular endothelial function, so in the subgroup analyses, we also sought to determine the effects of participants’ characteristics (age, basal BMI, and basal FMD). Our results showed that a worse basal FMD was associated with a larger improvement in FMD, while continuous aerobic exercise had no significant effect on FMD in people with normal basal FMD, which was consistent with a previous study, showing that when FMD was normal at baseline, there was no significant difference in the effect of the intervention on FMD, but those with impaired endothelial function may have greater improvement, and aerobic exercise would benefit individuals most in need of improved endothelial function (Swift et al., 2012; Ashor et al., 2015).

In atddition, our results showed that an older age was associated with a larger improvement in FMD, which was consistent with previous studies (Skaug et al., 2013; Konigstein et al., 2022). There is no doubt that endothelial function is affected by age (Ungvari et al., 2018). During aging, the vascular system produces excess superoxide and hydrogen peroxide, both of which inhibit the vasodilatory activity of NO and lead to the formation of free radicals (e.g., proximities), whereas aging results in a low-grade inflammatory phenotype in the vascular system (El Assar et al., 2013). The vascular endothelial function is impaired due to the oxidative stress increase and other factors, while aerobic exercise reduces oxidative stress by inhibiting oxidation and stimulating antioxidant pathways (Seals et al., 2019). Obesity is an independent predictor of CVDs, with progressive increases in BMI associated with increased risk of CVD (Hubert et al., 1983; Donato et al., 2018). Therefore, we divided the included studies based on their participants’ basal BMI into three groups: normal weight, overweight, and obese. Our results showed that compared with the control group, continuous aerobic exercise significantly improved FMD in normal weight, overweight, and obese people. In addition, a larger basal BMI was associated with larger improvements in FMD, indicating that continuous aerobic exercise had a better effect on FMD in higher BMI people than in lower BMI people. The concept that obesity is characterized by low levels of chronic and subclinical inflammation is becoming more widely accepted (Hajer et al., 2008). Excessive BMI produces high levels of inflammatory factors, and the pro-inflammatory state in adipose tissue leads to local insulin resistance and impaired inhibition of free fatty acids (FFA) release by insulin (Gustafson, 2010). Continuous aerobic exercise can reduce inflammatory factors and increase antioxidant capacity (Gielen et al., 2010; Vandercappellen et al., 2022). Aerobic exercise reduces BMI in obese individuals, and endurance training is associated with increased circulating high-density lipoprotein (HDL) levels and decreased very low-density lipoprotein (LDL) (Halverstadt et al., 2007). It improves insulin sensitivity (Newsom et al., 2013) and insulin signaling within the vascular endothelium promotes Akt-dependent phosphorylation and activation of eNOS to produce the vasodilator NO (Steinberg et al., 1994), thereby improving vascular endothelial function. In short, age and obesity are the main factors causing vascular endothelial dysfunction, and the different effects of continuous aerobic exercise to FMD can be proved by the intervention effect to participants with different age basal BMI.

### Limitations of the review

It is important to acknowledge some potential limitations of this meta-analysis. As for the inclusion criteria, all included studies were RCTs using continuous aerobic exercise interventions, which could not be completely blinded. As a result, subjective factors will contribute to a certain degree of deviation in quality evaluation. Additionally, there was a high degree of heterogeneity, which may be due to multiple risk factors such as smoking, food and alcohol intake, blood pressure, and blood lipids. Moreover, previous studies have shown that high-intensity intervals training (HIIT) can also be effective in improving FMD (Molmen-Hansen et al., 2012; Ghardashi et al., 2018; Boff et al., 2019), but our study only focused on the effect of continuous aerobic exercise on FMD, and future studies should take HIIT into account to make the results more comprehensive. Furthermore, gender differences were not considered due to the limited availability of separate male and female studies in this study. Finally, because of the significant heterogeneity in the results of the meta-analysis, the results of our study should be interpreted with caution.

## Conclusion

Our analysis indicated that continuous aerobic exercise, especially moderate-intensity and vigorous-intensity aerobic exercise, contributed to improving FMD. The effect of continuous aerobic exercise on improving FMD was associated with duration and participant’s characteristics. Specifically, a longer duration, an older age, a larger basal BMI, and a worse basal FMD contributed to more significant improvements in FMD.

## Data Availability

The original contributions presented in the study are included in the article/Supplementary Material, further inquiries can be directed to the corresponding authors.
